# Overcoming Recruitment Barriers in Urban Older Adults Residing in Congregate Living Facilities

**DOI:** 10.1155/2015/824672

**Published:** 2015-05-27

**Authors:** Adam Simning, Edwin van Wijngaarden, Yeates Conwell

**Affiliations:** ^1^Department of Psychiatry, University of Rochester, 300 Crittenden Boulevard, Rochester, NY 14642, USA; ^2^Department of Public Health Sciences, University of Rochester, 265 Crittenden Boulevard, Rochester, NY 14642, USA

## Abstract

*Background*. Participation of minority older adults in mental health research has been limited by mistrust, transportation difficulties, lack of knowledge, and insufficient community partnership. We describe strategies utilized to overcome these recruitment barriers. *Methods*. Our target population included 553 public housing residents of older adult high-rise buildings in Rochester, NY. We had a two-stage cross-sectional study: Stage 1 was a health survey for all residents and Stage 2 was a psychiatric interview of English-speaking residents aged 60 years and older. Recruitment occurred through mailings, onsite activities, and resident referrals. *Results*. Stage 1 had 358 participants (64.7% response) and Stage 2 had 190 (61.6% target population response), with higher participation among African Americans. We found some strategies effective for overcoming recruitment barriers. First, we partnered with a community agency and organized onsite educational activities to improve residents' trust. Second, the study occurred entirely onsite, which facilitated participation of functionally impaired residents. Third, onsite activities allowed the residents to learn about the study and complete surveys in person. Fourth, we provided immediate incentives that resulted in many study referrals. *Conclusions*. Although recruitment of minority older adults presents unique challenges, a multifaceted community-tailored approach mitigated several recruitment barriers in this mental health study.

## 1. Introduction

There is a great need to obtain better representation of diverse populations of older adults in mental health studies, and difficulty recruiting minority groups into research is well documented [1–3]. In response to the many barriers to recruitment, a science is slowly developing on how to increase participation of minority older adults in research. There remain, however, few concrete examples of strategies that can successfully recruit individuals from diverse populations into mental health studies.

Mistrust of medical researchers can be substantial, and a problematic issue for research recruitment is that African Americans—the second largest minority group in the United States [4]—do not trust doctors or medical research as much as others do [5]. There is a powerful history of mistreatment that has eroded African American trust in medical research [6, 7]. Among African Americans the term “medical research” triggered associations such as being lied to, corruption, deception, using people, and guinea pigs, and many had reservations about research scientists' motivations, which they thought were primarily a drive for money and prestige [8]. Such misgivings can be prominent in older African Americans because some may have witnessed or experienced medical research abuses [9, 10]. Some minority older adults may thereby feel uneasy in traditional research settings. To overcome mistrust, connecting with the community is a critical component of successful recruitment of minority older adults [11, 12], as is creating a common understanding between researchers and study participants [13, 14]. In particular, a key to recruiting underserved and hard-to-reach individuals is to involve people who are known and trusted within the community [15, 16]. Mistrust may also partly explain why solicitation methods that work well recruiting white older adults into research studies (e.g., advertisements) can have limited success with minority older adults [3, 17].

A second recruitment barrier is transportation difficulties. For many older adults, especially for those in minority groups which often are socioeconomically disadvantaged and tend to have worse health than whites [18], traveling to research sites can be difficult due to cost, illness, functional impairment, perceived inaccessibility of the research site, and security risks [7, 19]. Consequently, conducting research within the minority older adults' communities is one method to increase accessibility [3].

A third barrier to recruitment is that poor health literacy, limited mental illness knowledge, and not understanding the potential benefits of research participation can limit older adults from participating in research [3, 19–21]. In response, researchers have used community education projects, community-centered health fairs, and other venues to provide participants with knowledge regarding health- and research-related issues and to engage them with research staff [15, 19].

A fourth recruitment barrier is that relationships between the community and research institutions are often fleeting. Lack of meaningful feedback and little apparent concrete benefit of research to the community can foster reservations about investigators' motivations [8]. To strengthen community-academic relations, researchers can provide tangible benefits to the communities and maintain connections that may improve future research efforts within a community [12]. Disseminating findings back to the community also should be a component of community-based research [22] because it can help build lasting reciprocal relationships between researchers and the communities they study. To empower and more effectively engage communities, there is growing support for conducting community-based participatory research that represents a cooperative process in which both the researchers and community contribute to and learn from the research process [23].

In summary—although not necessarily unique to minority older adults or mental health research as recruitment of any sample of older adults can be difficult [19]—issues of mistrust, transportation difficulties, knowledge gaps, and a community-academic divide are considerable barriers limiting diverse populations of older adults from participating in mental health studies. Prior to the initiation of our two-stage mental health study, we devised a multifaceted recruitment approach that targeted these four anticipated recruitment barriers with strategies based on recommendations derived from prior recruitment efforts. Our objective here was to describe resident response to our recruitment strategies as a means of characterizing their apparent effectiveness. Our analytic goals were to estimate the study's response and characterize the demographic differences between responders and nonresponders. We hypothesized that nonresponders were more likely to be older, male, of African American race, and of Hispanic ethnicity. There are few empirical examples of how to recruit a diverse population of older adults into mental health studies, and this study's findings aim to provide guidance for future research efforts seeking to do so.

## 2. Materials and Methods

### 2.1. Study Setting

From May 2009 to June 2010, our two-stage cross-sectional epidemiologic study occurred within four public housing high-rises reserved for residents aged 50 years and older in Rochester, NY. Residents had a median age of 64.4 years and 46.8% were male, 24.8% were Hispanic, and 61.0% were African American; non-Hispanic whites only comprised 11.8% of the residents. The high-rise buildings housed between 73 in the smallest building and 299 public housing residents in the largest building and had differing ethnic (5.5% to 40.1% Hispanic) and racial (46.0% to 82.7% African American) compositions. The study's primary goals were to use a psychiatric research interview to characterize anxiety, depression, and service utilization among English-speaking residents aged 60 years and older. These findings will serve as a basis for better matching mental health services with need for such services.

### 2.2. Study Design

The study had two stages that corresponded to a health survey, which occurred in Stage 1 of the study, and the subsequent psychiatric research interview that comprised Stage 2. Stage 1's sampling frame consisted of all public housing residents, whereas Stage 2 targeted English-speaking residents aged 60 years and older with capacity to provide informed consent. Our recruitment was limited in that the Rochester Housing Authority did not permit us to initiate contact through telephone or door-to-door solicitation because it did not want the study to intrude upon residents uninterested in participating. The primary investigator (P.I.), a non-Hispanic white M.D./Ph.D. student, was responsible for all aspects of data collection and had received psychiatric research interview training through the University of Rochester's Psychiatry Department. Due to the limited research staff, data collection proceeded in one high-rise building at a time; the same recruitment strategies, however, were employed at each high-rise. The University of Rochester Human Subjects Review Board approved this study.

#### 2.2.1. Stage 1

During the first stage, we sent mailings to all residents. Every mailing was translated using a professional translating service with Hispanic residents receiving mailings in both English and Spanish. Prior to study recruitment, Hispanic ethnicity was determined by the public housing demographic information that was provided to the study team. In general, the mailed surveys were rarely returned to the study team and most recruitment occurred in person. The objectives of Stage 1 were to engage residents and facilitate recruitment into Stage 2 as well as to collect health data that would allow for comparisons across demographic groupings not represented in Stage 2 (e.g., residents younger than 60 years). The first two mailings introduced the study and provided educational material on healthy aging and cognitive health, topics we thought would be relevant and interesting to this older adult community. More specifically, the first mailing addressed some aging myths (e.g., “To be old is to be sick,” “You can't teach an old dog new tricks”) while the second mailing had 10 tips for supporting memory such as “Exercise your body” and “Keep in touch [socially].” The 3rd mailing contained several questions on demographic information and a brief four-question health survey [24]; we made this mailing as brief as possible to maximize resident response. After the first round of Stage 2 research interviews, Stage 1 nonresponders received another health questionnaire mailing. On the Stage 1 survey, residents indicated their willingness to participate in Stage 2 via a “Yes/No” response to a question asking them if they would be interested in answering more questions about their health and participating in a follow-up study; residents endorsing “Yes” provided their phone number so that we could call them and schedule an interview. Several half-day onsite educational activities and recruitment sessions (conducted in English) coincided with each of the educational and health survey mailings, respectively. The onsite educational activities mirrored the content of the mailings (e.g., after the first mailing, a table was set up in the high-rise lobby where study staff would interact with residents and discuss the mailings). After completion of the study in each building, residents received a thank you letter summarizing the health survey results. There were no exclusion criteria for Stage 1. Most participants immediately received $5 when they turned in the health survey to the P.I. in person, while a few returned the survey by mail or gave it to the onsite social worker.

#### 2.2.2. Stage 2

The second stage included Stage 1 respondents who were 60 years or older and English-speaking (non-English speakers were excluded) and had capacity to provide informed consent. Determination of capacity to provide informed consent occurred in person prior to commencing the research interview; we evaluated capacity for consent with a series of questions that assessed the participants' understanding of the study and excluded residents unable to adequately answer these questions. Due to the sensitive nature of psychiatric interviews, we did not utilize proxy informants. Literacy, medical comorbidities, and psychiatric comorbidities were not exclusion criteria. Stage 2 comprised a 1.5-hour interview and assessed anxiety and depression with the Structured Clinical Interview for the DSM-IV [25]. The interviews were in person and primarily occurred in the residents' apartments or other private onsite high-rise locations. Participants promptly received $25.

### 2.3. Recruitment Barrier Strategies

In our recruitment approach many strategies addressed recruiting issues relating to mistrust, transportation, lack of knowledge, and the community-academic divide. Although some strategies targeted multiple barriers, we have paired each strategy with the barrier it primarily targeted to avoid redundancy. We adopted three strategies to address the mistrust barrier: (1) partnered with a community services agency familiar to residents; (2) sent informational mailings; and (3) organized onsite educational activities. Our strategy for overcoming the transportation barrier was to conduct the study entirely within the high-rises. To address the lack of knowledge barrier, we relied on three strategies: (1) a series of recruitment mailings, (2) onsite recruitment sessions, and (3) attendance at resident council meetings. Lastly, we had four strategies to overcome the community-academic divide barrier: (1) immediately reimbursed residents $5 for the health survey and $25 for the psychiatric interview; (2) attempted to limit the interview's burdensomeness/length; (3) mailed residents a summary of the health survey findings and thanked them for participating; and (4) presented the findings to the resident council meetings, aging services agency assisting the residents, and Rochester Housing Authority.

### 2.4. Statistical Analysis

Basic descriptive statistics described the residents' demographics. We reported medians and interquartile ranges for variables not normally distributed. Bivariate analyses characterized the sociodemographic differences between responders and nonresponders. The Pearson chi-square test examined differences of categorical variables, while the nonparametric Wilcoxon rank sum test for nonnormal data contrasted differences in continuous variables. A *p* value of 0.05 or less indicated statistical significance, and we conducted analyses with SAS statistical software version 9.2 (SAS Institute, Inc., Cary, NC).

## 3. Results

### 3.1. Stage 1 Response

Stage 1 target sample was all public housing high-rise residents (*n* = 553), and most residents (*n* = 358; 64.7%) completed the health survey (Table 1). Non-Hispanics, African Americans, and women were in the majority, comprising 80.4%, 64.8%, and 55.3% of Stage 1 responders. While there were no age or gender differences between responders and nonresponders, Stage 1 responders were more likely to be of African American race and non-Hispanic ethnicity. Among residents completing Stage 1 brief health survey, 329 (91.9%) expressed an interest in participating in Stage 2 psychiatric interview.

### 3.2. Stage 2 Response

Our Stage 2 target sample consisted of non-Hispanic residents aged 60 years and older who were English-speaking and cognitively able to provide informed consent (*n* = 292), of whom we interviewed 180 (61.6%) (Table 2). Among non-Hispanic residents, Stage 2 responders were younger than nonresponders, but the response did not vary by gender or race.

We excluded Hispanic residents from Stage 2's response rate denominator because an unknown majority of Hispanic residents were not fluent in English: of Stage 1 Hispanic responders, 41.4% reported being able to speak English (many of these were not fluent), and we were only able to interview 10 Hispanic Stage 1 responders for a total of 190 interviewees.

### 3.3. Building-Specific Response Rates

Stage 1 response rates (included Hispanic residents in the denominator) varied from a low of 60.9% at the largest and most diverse building (*n* = 299 residents; 40.1% Hispanic; 46.0% African American) to a high of 75.3% at high-rise number 4, which had many fewer residents and a more homogenous population (*n* = 81; 6.2% Hispanic; 82.7% African American). Stage 2 building-specific response rates (did not include Hispanic residents in the denominator) mirrored those in Stage 1 (Table 3).

### 3.4. Overcoming Mistrust

To address mistrust, we adopted three strategies. The first strategy was to partner with an aging services provider that for many years has employed onsite social workers that provided assistance to residents. This partnership allowed us to send 4 to 5 mailings to all residents that were signed by us and the building-specific social worker. This linkage with the aging services agency enabled residents to discuss concerns they had regarding the study with the social workers and also allowed the social worker to introduce us to residents.

The second strategy for overcoming mistrust was to use a series of informational mailings on healthy aging and cognitive health (described previously in Section 2). These initial mailings provided a service to the residents and acquainted many of them with us.

The third strategy was to organize educational activities in the high-rise lobbies that coincided with each mailing. Placing the educational activities in the lobbies generated much more resident contact than did having the activities in community rooms, which were often removed from the buildings' daily traffic. The onsite educational activities spanned two days, took place in either the mornings or afternoons, and contained free nutritious snacks, which facilitated interaction with many residents and helped build rapport. Because of these onsite activities, residents became accustomed to seeing the P.I. in the high-rise and many grew to interact with him on a friendly and casual level. Such residents would often later participate and actively recruit other residents into the study. Many residents were initially hesitant or neutral regarding participation, and when trusted residents endorsed our study, recruitment surged as knowledge and acceptance of the study spread through the public housing social networks.

### 3.5. Overcoming Lack of Transportation

We had one strategy to overcome recruitment difficulties related to transportation, which was to situate the study entirely within the high-rises and require no traveling by study participants. Bringing the study to the community was necessary because the residents were socioeconomically distressed and many had functional impairments that limited their mobility. Some residents also expressed strong reservations about traveling in winter due to safety concerns related to icy conditions. Conducting the psychiatric research interview in a convenient place and time helped convince many ambivalent residents to participate (e.g., some residents would only participate in an interview after returning home from their work; others were only willing to participate if the interview did not overlap with their favorite TV shows).

### 3.6. Overcoming the Knowledge Gap

Based on our interactions with the residents and feedback from onsite social workers, limited education, illiteracy, and lack of knowledge regarding mental illness were highly prevalent among the high-rise residents. To address the lack of knowledge recruitment barrier, we relied on three strategies: (1) a series of recruitment mailings, (2) onsite recruitment sessions, and (3) attendance at resident council meetings.

The series of recruitment mailings received a mixed reaction from the residents. While some residents read the mailings, and a few talked with us or the onsite social worker about the mailings, a large proportion of residents also did not read them: we saw many unopened mailings thrown away or in resident apartments during Stage 2 interviews. Of note is that a few residents voiced suspicion about the mass mailings due to the numerous scams that had targeted them.

The onsite recruitment sessions allowed us to interact verbally with residents and assist them in completing the health survey, which was particularly important for the many residents unable to read. Residents often had many questions about the study and these recruitment sessions did much to alleviate concerns and to increase knowledge of the study.

Throughout the course of the study, the P.I. attended some building-specific resident council meetings to introduce himself, discuss the study, and answer resident questions. After attending these meetings, some residents became friendlier and made a greater effort to refer others to the study.

### 3.7. Overcoming the Community-Academic Divide

We addressed the last recruitment barrier—that of community-academic relationship building issues—in four ways. First, we immediately reimbursed residents for the health survey and psychiatric interview. These reimbursements helped ensure that the study was of tangible benefit to the participants, garnered support for our study, and resulted in referrals as many participants actively recruited others.

Second, to foster community support of our research and minimize its possible negative impact, we attempted to limit the burdensomeness of the interview. If the interview was too burdensome, it may have discouraged residents from participating in this study and possibly in future research efforts, whereas a less exhaustive interview could have the opposite effect. Consequently, the median interview duration was 94 minutes, with an interquartile range of 77 to 119 minutes. Although we tried to have the interview be relatively brief, and this interview duration was well below other psychiatric epidemiology studies that had interviews lasting beyond 2 hours [26, 27], some interviewees—relatively many of whom were male—would have preferred a shorter interview.

Third, after we completed the interviews in a high-rise, the residents received a mailing thanking them for their participation and summarizing the health survey findings for their building.

Fourth, we presented the findings to the resident council meetings, aging services agency assisting the residents, and Rochester Housing Authority. In support of this strategy is that throughout the study many residents expressed a desire to learn about the study findings.

## 4. Discussion

### 4.1. Study Response

Achieving a robust response among diverse urban older adults can be challenging as participation in epidemiologic studies has been declining [28] and many urban older adults are African Americans, a group for which recruitment into research has been difficult [7]. In consideration of this environment, our response rate of 61.6% for the non-Hispanic target population is satisfactory and compares favorably with other public housing studies [15, 29, 30]. Importantly, we were able to achieve a 60% response rate without directly soliciting residents by cold-calling or knocking. These direct solicitation recruitment methods were used in prior public housing studies [10, 15, 30, 31] and national psychiatric epidemiology studies, which achieved a 70% response following multiple contacts of nonresponders and high monetary incentives [32]. Rather we organized onsite educational activities and recruitment sessions at which residents primarily approached us.

There were differences between responders and nonresponders to Stage 1 health survey and Stage 2 interview that partly supported our hypotheses: Stage 1 respondents were more likely to be non-Hispanic and African American, whereas Stage 2 interviewees were more likely to be younger (consistent with prior studies having younger respondents [33]) and only 5.3% were Hispanic. Stage 1's ethnicity difference suggests that the Spanish-translated mailings were insufficient to overcome the language barrier, and it is possible that many of the Spanish-speaking residents were also illiterate (as were many of the non-Hispanic residents). Having a Spanish-speaking researcher at the recruitment activities to facilitate interaction and rapport-building with Hispanic and Latino residents would have been highly beneficial. Overall, our approach to accommodating the non-English speakers seemed to be only partially effective in Stage 1 and was absent in Stage 2 because of study resource limitations.

Response also varied across buildings. The largest building had the worst response rate, which was likely due to two factors. First, at times the demand to participate in Stages 1 and 2 was too much for us to accommodate. In the largest building it took many months to complete the interviews and it was difficult to maintain momentum as some Stage 1 respondents forgot about the study before they were contacted to schedule a Stage 2 interview. Second, 4 in 10 of the largest building residents were Hispanic, the majority of whom was not fluent in English. This made it difficult for the English-speaking P.I. to have meaningful in-person interactions with a large portion of the residents. In contrast, the building with the best recruitment (high-rise number 4) was relatively small and had far fewer non-English speakers. The varied responses across building sites suggest that researchers should be flexible in their recruitment approach to cater to the different community cultures present even within a single target population. While one research staff member was adequate in some situations, additional bilingual research staff would have been very useful in other settings.

### 4.2. Recruitment Barriers Summary

We anticipated that many residents would have considerable misgivings towards university researchers. Our efforts to partner with a community agency and provide an educational service to the residents seemed to be partially successful in building residents' trust as many residents—some of whom were initially hesitant and skeptical about our motivations—became quite friendly. A cautionary note must be raised, however: each high-rise had unique internal politics resulting in different resident cliques, and courting of one could harm recruitment from other cliques. Issues such as these may explain how differing recruitment approaches can influence the outcomes observed [34]. A practical approach to limit such biased outcomes would be to use multiple recruitment approaches that cast a wider net for engaging a variety of individuals (e.g., mailings, fliers, in-person recruitment, and referrals by community agencies). Our study would have likely had more participation if we formally involved the onsite social workers, building staff, resident council members, and study participants in recruitment.

Bringing the study to the residents was probably our most important recruitment strategy. Analogous strategies may be applied in communities for which residents reside in their own houses (e.g., recruitment from places where the residents congregate such as churches and senior nutrition centers).

In acknowledgement of the community-academic divide, we sought to balance the benefit to and demand upon the community by providing monetary incentives and limiting the interview burdensomeness. Most seemed happy to have participated and many were eager to participate in future research efforts. Nonetheless, there were a few who expressed annoyance with the interview length for whom we could have had available an abbreviated interview containing the key study constructs.

### 4.3. Limitations and Strengths

Our findings have a number of limitations to consider. First, this study was based in a single geographic locale with recruitment strategies designed to take advantage of the congregate living setting, which may limit its generalizability. Second, the study utilized one recruitment approach for all residents, thereby preventing a direct comparison of effectiveness across recruitment strategies. Third, we did not systematically use feedback from building residents in our evaluation of recruitment strategies (e.g., no formal qualitative interviews); rather we used unsolicited resident feedback to inform our narrative. Fourth, we had limited information on study nonresponders, which weakened our ability to characterize them. Fifth, due to the limited resources, a non-Spanish-speaking white doctoral student conducted the educational activities, recruitment efforts, and psychiatric research interviews. Although matching recruitment staff on race and ethnicity is ideal if possible, white and African American researchers can have similar recruitment yields among minority populations if properly trained [35]. Due to language barriers, however, our study had limited accessibility to the 24.8% of the residents who were Hispanic.

The study recruitment approach has some notable strengths. In less than 1.5 years we were able to collect general health information from 358 and detailed mental health and service utilization data from 190 residents. This study's multifaceted recruitment approach achieved a 60% response rate in spite of being limited by the inability to use unsolicited telephone and at-home recruitment. Furthermore, study responders had a larger proportion of African Americans than study nonresponders, which is encouraging as older African Americans are usually underrepresented in research studies. The study also relied on relatively few resources (training grants funded the study) and may be easily adapted to other research studies.  Table 4 summarizes our recommendations for studies seeking to recruit urban older adults, especially those living in congregate settings, and we recommend that future studies formally evaluate our impressions regarding these recruitment strategies.

## 5. Conclusions

Our study and others [15] suggest that socioeconomically disadvantaged older adults living in urban congregate settings are receptive to research that concurrently combines research efforts with educational outreach to assist the individuals. Among these urban older adults, many of whom are racial minorities, the willingness and need to participate in research exist, but the impetus is on researchers to leave their academic institutions and meet these individuals in the communities in which they reside.

## Figures and Tables

**Table 1:** Stage 1 responders and nonresponders by sociodemographic groupings.

Demographics	Nonresponders		Responders		*p* value^*∗*^
	*n*	% or median (interquartile range)	*n*	% or median (interquartile range)	
Age	195	66.0 (59.0 to 74.0)	358	64.0 (59.0 to 70.8)	0.077
Gender					0.171
Female	96	49.2	198	55.3	
Male	99	50.8	160	44.7	
Ethnicity					<0.001
Hispanic	67	34.4	70	19.6	
Non-Hispanic	128	65.6	288	80.4	
Race					0.012
African American	104	53.9	232	64.8	
Non-African American	89	46.1	126	35.2	

^*∗*^ *p* values determined by the nonparametric Wilcoxon two sample tests, Pearson chi-square tests, or Fisher's exact test.

**Table 2:** Stage 2 responders and nonresponders by sociodemographic groupings.

Demographics	Nonresponders		Responders		*p* value^*∗*^
	*n*	% or median (interquartile range)	*n*	% or median (interquartile range)	
Age	112	71.5 (65.0 to 79.0)	180	66.3 (63.1 to 72.8)	<0.001
Gender					0.214
Female	57	50.9	105	58.3	
Male	55	49.1	75	41.7	
Race					0.202
African American	88	78.6	152	84.4	
Non-African American	24	21.4	28	15.6	

^*∗*^ *p* values determined by the nonparametric Wilcoxon two sample tests, Pearson chi-square tests, or Fisher's exact test.

**Table 3:** Stage 1 and Stage 2 building-specific response rates.

	High-rise number 1		High-rise number 2		High-rise number 3		High-rise number 4		Total	
	*n*	%	*n*	%	*n*	%	*n*	%	*n*	%
Stage 1 survey response	47	64.4	68	68.0	182	60.9	61	75.3	358	64.7
Stage 2 interview response^*∗*^	30	60.0	46	61.3	65	57.0	39	73.6	180	61.6

^*∗*^Total eligible for Stage 2 includes public housing residents who were non-Hispanic, English-speaking, 60 years or older, and cognitively able to provide informed consent; we excluded Hispanic residents from Stage 2's response rate denominator because an unknown majority of Hispanic residents were not fluent in English.

**Table 4:** Recruitment recommendation summary.

(1) Partner with known and trusted community agencies.	

(2) Perform study at community sites accessible to and frequently visited by the study target population.	

(3) Prior to recruitment, conduct a series of educational/outreach activities to acquaint the community with research staff.	

(4) Provide immediate reimbursement to generate enthusiasm for and increase referrals into the study.	

(5) To the extent feasible, limit the research burden upon participants.	

(6) Use a wide variety of recruitment strategies to limit sampling bias and improve recruitment rates.	

(7) Have research staff that is fluent in the study target population's language(s).^*∗*^	

(8) Share study findings with participants.	

^*∗*^Our study did not do this and we were unable to adequately recruit Hispanic residents.

## References

[1] Shavers-Hornaday V. L., Lynch C. F., Burmeister L. F., Torner J. C. (1997). Why are African Americans under-represented in medical research studies? Impediments to participation. *Ethnicity and Health*.

[2] Gavaghan H. (1995). Clinical trials face lack of minority group volunteers. *Nature*.

[3] Areán P. A., Gallagher-Thompson D. (1996). Issues and recommendations for the recruitment and retention of older ethnic minority adults into clinical research. *Journal of Consulting & Clinical Psychology*.

[4] U.S. Census Bureau (2014). *Profile of General Population and Housing Characteristics: 2010*.

[5] Corbie-Smith G., Thomas S. B., George D. M. M. S. (2002). Distrust, race, and research. *Archives of Internal Medicine*.

[6] Wasserman J., Flannery M. A., Clair J. M. (2007). Rasing the ivory tower: the production of knowledge and distrust of medicine among African Americans. *Journal of Medical Ethics*.

[7] Qualls C. D. (2002). Recruitment of African American adults as research participants for a language in aging study: example of a principled, creative, and culture-based approach.. *Journal of Allied Health*.

[8] Corbie-Smith G., Thomas S. B., Williams M. V., Moody-Ayers S. (1999). Attitudes and beliefs of African Americans toward participation in medical research. *Journal of General Internal Medicine*.

[9] Earl C. E., Penney P. J. (2001). The significance of trust in the research consent process with African Americans. *Western Journal of Nursing Research*.

[10] Dennis B. P., Neese J. B. (2000). Recruitment and retention of african american elders into community-based research: lessons learned. *Archives of Psychiatric Nursing*.

[11] Curry L., Jackson J. (2003). The science of including older ethnic and racial group participants in health-related research. *The Gerontologist*.

[12] Moreno-John G., Gachie A., Fleming C. M. (2004). Ethnic minority older adults participating in clinical research: developing trust. *Journal of Aging & Health*.

[13] McCallum T. J., Arlien C. R. (2006). Enhancing the matching model of recruitment through focus groups. *Aging & Mental Health*.

[14] Levkoff S., Sanchez H. (2003). Lessons learned about minority recruitment and retention from the centers on minority aging and health promotion. *The Gerontologist*.

[15] Jeffries S. K., Choi W., Butler J., Harris K. J., Ahluwalia J. S. (2005). Strategies for recruiting African-American residents of public housing developments into a randomized controlled trial. *Ethnicity & Disease*.

[16] Henderson J. N., Gutierrez-Mayka M., Garcia J., Boyd S. (1993). A model for Alzheimer's disease support group development in African-American and Hispanic populations. *The Gerontologist*.

[17] Bistricky S. L., MacKin R. S., Chu J. P., Areán P. A. (2010). Recruitment of African Americans and Asian Americans with late-life depression and mild cognitive impairment. *The American Journal of Geriatric Psychiatry*.

[18] Williams D. R. (1998). African-American health: the role of the social environment. *Journal of Urban Health*.

[19] Ballard E. L., Nash F., Raiford K., Harrell L. E. (1993). Recruitment of black elderly for clinical research studies of dementia: the CERAD experience. *The Gerontologist*.

[20] Wolf M. S., Gazmararian J. A., Baker D. W. (2005). Health literacy and functional health status among older adults. *Archives of Internal Medicine*.

[21] Kutner M., Greenberg E., Jin Y., Paulsen C. (2003). *The Health Literacy of America's Adults: Results from the 2003 National Assessment of Adult Literacy*.

[22] Stahl S. M., Vasquez L. (2004). Approaches to improving recruitment and retention of minority elders participating in research: examples from selected research groups including the National Institute on Aging's Resource Centers for Minority Aging Research. *Journal of Aging & Health*.

[23] Leung M. W., Yen I. H., Minkler M. (2004). Community-based participatory research: a promising approach for increasing epidemiology's relevance in the 21st century. *International Journal of Epidemiology*.

[24] Moriarty D. G., Zack M. M., Kobau R. (2003). The Centers for Disease Control and Prevention's Healthy Days Measures—population tracking of perceived physical and mental health over time. *Health and Quality of Life Outcomes*.

[25] First M. B., Spitzer R. L., Gibbon M., Williams J. B. W. (2002). *Structured Clinical Interview for DSM-IV-TR Axis I Disorders*.

[26] Jackson J. S., Torres M., Caldwell C. H. (2004). The National Survey of American Life: a study of racial, ethnic and cultural influences on mental disorders and mental health. *International Journal of Methods in Psychiatric Research*.

[27] Kessler R. C., Berglund P., Chiu W. T. (2004). The US National Comorbidity Survey Replication (NCS-R): design and field procedures. *International Journal of Methods in Psychiatric Research*.

[28] Galea S., Tracy M. (2007). Participation rates in epidemiologic studies. *Annals of Epidemiology*.

[29] Rabins P. V., Black B. S., Roca R. (2000). Effectiveness of a nurse-based outreach program for identifying and treating psychiatric illness in the elderly. *The Journal of the American Medical Association*.

[30] Robison J., Schensul J. J., Coman E. (2009). Mental health in senior housing: racial/ethnic patterns and correlates of major depressive disorder. *Aging and Mental Health*.

[31] Rabins P. V., Black B., German P. (1996). The prevalence of psychiatric disorders in elderly residents of public housing. *The Journals of Gerontology, Series A: Biological Sciences and Medical Sciences*.

[32] Heeringa S. G., Wagner J., Torres M., Duan N., Adams T., Berglund P. (2004). Sample designs and sampling methods for the Collaborative Psychiatric Epidemiology Studies (CPES). *International Journal of Methods in Psychiatric Research*.

[33] Michelet M., Lund A., Sveen U. (2014). Strategies to recruit and retain older adults in intervention studies: a quantitative comparative study. *Archives of Gerontology and Geriatrics*.

[34] Izal M., Nuevo R., Montorio I., Pérez-Rojo G. (2009). Method of recruitment and the scores of self-report measures: the example of worry in the elderly. *Archives of Gerontology and Geriatrics*.

[35] Thompson E. E., Neighbors H. W. (1996). Recruitment and retention of African American patients for clinical research: an exploration of response rates in an urban psychiatric hospital. *Journal of Consulting & Clinical Psychology*.

